# Effects of High-Intensity Interval Training on Muscle Strength for the Prevention and Treatment of Sarcopenia in Older Adults: A Systematic Review of the Literature

**DOI:** 10.3390/jcm13051299

**Published:** 2024-02-25

**Authors:** José Alfonso Morcillo-Losa, Maria del Pilar Díaz-Martínez, Halil İbrahim Ceylan, Beatriz Moreno-Vecino, Nicola Luigi Bragazzi, Juan Párraga Montilla

**Affiliations:** 1 Department of Didactics of Corporal Expression, University of Jaén, 23071 Jaén, Spain; jamlosa@ujaen.es (J.A.M.-L.); pilardm@gmail.com (M.d.P.D.-M.); jparraga@ujaen.es (J.P.M.); 2 Physical Education and Sports Teaching Department, Kazim Karabekir Faculty of Education, Ataturk University, 25030 Erzurum, Turkey; 3 Department of Physical Activity and Sport Sciences, Centre d’Ensenyament Superior Alberta Giménez CESAG, Pontifical University of Comillas, 07013 Palma, Spain; bmvecino@comillas.edu; 4 Laboratory for Industrial and Applied Mathematics (LIAM), Department of Mathematics and Statistics, York University, Toronto, ON M3J 1P3, Canada; 5 Human Nutrition Unit (HNU), Department of Food and Drugs, Medical School, University of Parma, 43125 Parma, Italy

**Keywords:** sarcopenia, older adults, aging, high-intensity interval training, muscular strength

## Abstract

Sarcopenia is a significant health concern primarily affecting old adult individuals, characterized by age-related muscle loss, and decreased strength, power, and endurance. It has profound negative effects on overall health and quality of life, including reduced independence, mobility, and daily activity performance, osteoporosis, increased fall and fracture risks, metabolic issues, and chronic diseases like diabetes and cardiovascular conditions. Preventive strategies typically involve a combination of proper nutrition and regular physical activity. Among strength training exercises, high-intensity interval training (HIIT) stands out as the most effective approach for improving muscle function in older adults with sarcopenia. The current review identifies and summarizes the studies that have examined the effects of HIIT on muscle strength in older adults as an element of the prevention and treatment of sarcopenia. A systematic search using several computerized databases, namely, MEDLINE/PubMed, Scopus, SPORTDiscus, and Web of Science, was performed on 12 January 2023, according to the Preferred Reporting Items for Systematic Reviews and Meta-Analyses (PRISMA) guidelines. A total of 224 studies were initially retrieved. A total of five studies met the selection criteria. HIIT training shows improvements in body composition and functional and cardiorespiratory capacity, has benefits on muscle strength, increases muscle quality and architecture, and is associated with muscle hypertrophy in healthy older adults. Nonetheless, given the shortcomings affecting primary research in terms of the limited number of studies and the high risk of bias, further research is warranted.

## 1. Introduction

**Figure 1:**
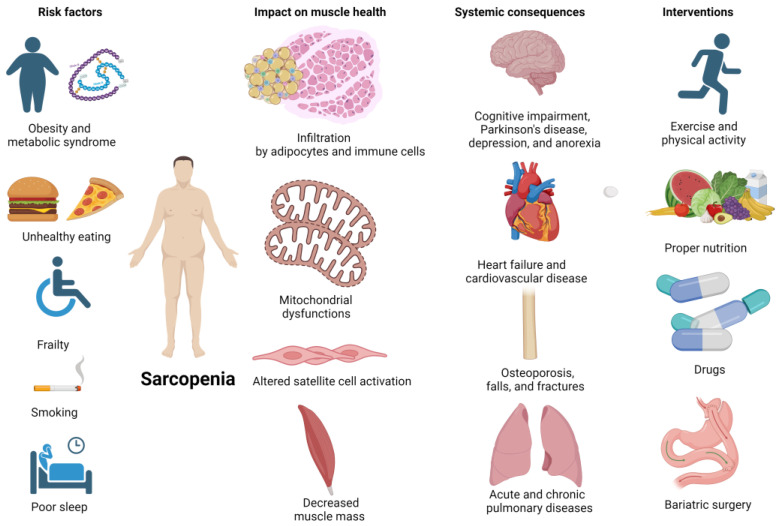
Sarcopenia, its risk factors, its impact on muscle health, systemic consequences, and potential interventions (Created with BioRender.com, accessed on 28 January 2024).

Sarcopenia, defined as age-related loss of muscle mass and function (i.e., strength, power, and endurance), represents a concerning medical issue that usually affects older adults, dramatically impacting their health and quality of life [1,2]. Its risk factors include unhealthy lifestyle behaviors and underlying comorbidities, while its detrimental effects include limited independence, mobility, and ability to perform daily activities, decreased bone density resulting in osteoporosis and increased risk of falls and fractures, metabolic impairments (such as insulin resistance, obesity, diabetes, and metabolic syndrome), and chronic disease like cardiovascular disease [3]. The management of people with sarcopenia can be challenging and early detection and intervention is paramount in minimizing its negative impacts. Preventive programs usually adopt multipronged approaches, combining regular physical activity, and strength training exercises, and ensuring adequate nutrition [4]. Currently, the most optimal and effective treatment for sarcopenia in older adults is strength training, which has been found to be more effective than other nutritional, hormonal, and pharmacological interventions in increasing muscle function [5] (Figure 1). 

High-intensity interval training (HIIT) has gained increasing popularity in recent years: it is characterized as having discontinuous, alternating periods of short-term (from 6 s to 4 min) work, at an intensity > 80–85% of the maximum heart rate (MHR) and maximal oxygen uptake (VO_2_max), scoring eight on the modified Borg Scale (on a scale from 0 to 10), and recovery periods of 1–5 min, at a lower intensity (60% MHR). These work periods are repeated several times [6], with an average duration of the programs of 12 to 16 weeks [7]. There is evidence for the efficacy of HIIT in adults over the age of 70, but studies are scarce when subjects are over the age of 90. 

HIIT has previously been shown to be safe, feasible, and effective in patients with diabetes, heart failure, and coronary artery disease [8,9], increasing muscle function as measured by the “European Working Group on Sarcopenia in Older People” (EWGSOP2), and improving physical performance in older adults [1,10,11,12].

The benefits of HIIT on muscle quantity and quality remain unclear, although significant improvements are reported, with varying results depending on the technique used for measurement [13]. Despite these potential benefits on strength, it would be inappropriate to implement HIIT with an exclusive focus on resistance training, as it must also be accompanied by aerobic, flexibility, and balance training to obtain maximum benefits [1]. 

Resistance training is based on the demand for strength in order to request an activation of muscle contraction. It is an effective method to combat the pernicious effects of sarcopenia, both structurally (i.e., by stimulating hypertrophy) and functionally (by increasing anaerobic endurance, muscular strength, and improving neuromuscular adaptation and physical performance) [14]. High-intensity strength exercises at 80% of 1RM have the greatest benefits in improving muscle strength and size, showing increases of 11% in muscle area, 34% in type I fibers, and 28% in type II fibers, with a decrease in body fat, and an increase in bone mineral density and oxygen consumption, as well as an optimization of glucose uptake and utilization [15]. In reference to aerobic training, HIIT is located in the anaerobic phase or the metabolic instability phase [16,17]. It is used for the prevention and treatment of sarcopenia, and improves balance, strength, muscle mass, and muscular endurance [18]. The increase in time and intensity should be methodologically progressive [19]. There is currently scientific evidence that mechanical vibration training can be effective in enhancing muscle strength, and this increase is linked to inter and intramuscular coordination. The attractiveness of applying it to frail people is that it involves less effort, can be applied with low impact, and requires less motor competence. 

Regarding the recommended effects and optimal doses of resistance training in older adults, the following five primary components should be applied for proper training control in HIIT sessions [20]: (i) interval intensity, controlled by MHR, reserve heart rate (RHR), rated perceived exertion (RPE), or maximum aerobic speed (MAS); (ii) duration of the interval, usually from 90 s to 150 s, properly adjusted to the MAS; (iii) (active and short) recovery intensity of 60% maximum frequency; (iv) recovery duration, based on RPE and the coach experience; and (v) number of intervals, depending on the exercise intensity and the person’s physical condition. There is a consensus on a duration of >10 min at 95% VO_2_max.

The main physiological responses and adaptations of HIIT at the level of the neuromuscular system consist of the progressive participation of all motor units of fibers type I, IIa, and IIx, by an oxidative metabolism with high cytosolic glycolytic involvement and a progressively acidotic internal cellular environment. These characteristics make HIIT beneficial for the aging process [7]. In the context of high exercise intensities inherent to HIIT, both respiratory rate and tidal volume experience an augmentation. Minute ventilation, representing the total volume of air breathed in one minute, can reach and even exceed 150 L per minute during high-intensity exercise, which is notably 17 times higher than the resting values [7,16]. 

The most prominent response and adaptation within the neuroendocrine system is associated with the sympathetic-adrenal system, which takes precedence in directing the endocrine activity during exercise by supporting catecholamines. This results in hypertrophy of the adrenal gland, augmenting its catecholamine content and enabling individuals to sustain intense exertion for extended periods. The highest adrenal stimulation occurs during the repetition of very intense exercise, characteristic of HIIT. This heightened stimulation leads to maximum levels of muscle and liver glycogenolysis, with near-maximal activation of phosphorylase [7,16].

In the context of oxygen consumption, the objective during phase III intensity is to elevate its maximum values. This parameter is contingent on genetic factors and can be enhanced by up to 20% through training, by augmenting the oxygen extraction capacity of the active muscles. This, in turn, results in a decreased oxygen capacity of the involved muscles, attributed to the high speed of shortening and tension development. The responsibility for supplying oxygen to compensate for the low tissue oxygen pressure falls on myoglobin. Additionally, the induction of hypoxia is employed to further enhance the muscle’s oxidative capacity [7,16]. 

The distinctive feature of HIIT is its short duration compared to continuous and prolonged moderate-intensity training, yielding similar improvements in terms of cardiovascular fitness, and demonstrating greater benefits in maximal aerobic capacity. Furthermore, HIIT enhances the oxidative capacity of muscles and increases the recruitment of muscle fibers, engaging a greater number of muscle fibers. Remarkably, just six HIIT sessions over a two-week period can enhance the metabolic control mechanisms and the activity of key mitochondrial enzymes, such as citrate synthase and cytochrome oxidase [21]. 

Physical exercise plays a pivotal role in the prevention and treatment of pathologies commonly observed in older adults, particularly sarcopenia. Currently, there is significant debate among researchers regarding the most beneficial type of training program for maintaining a good quality of life in older adults. Given the limited evidence available on HIIT in this specific population, there is a pressing need for further research to fill this gap in knowledge. Therefore, the present systematic review was undertaken with the aim of providing additional evidence and insights into the existing body of knowledge. 

## 2. Materials and Methods

### 2.1. Study Design and Protocol

The strategy for conducting the present systematic review strategy was compliant with the “Preferred Reporting Items for Systematic Reviews and Meta-analyses” (PRISMA) guidelines [22]. The protocol was registered within the “International Platform of Registered Systematic Review and Meta-Analysis Protocols” (code number INPLASY202310069, approval date 20 January 2023).

### 2.2. Eligibility Criteria 

The inclusion and exclusion criteria are outlined in Table 1, structured according to the “Population, Intervention, Comparison/Comparator, Outcome, Study Design” (PICOS) approach. This systematic framework provides a clear and organized delineation of the parameters governing the selection of studies. Adhering to the PICOS criteria ensures methodological consistency and clarity in defining the characteristics essential for study eligibility, promoting a systematic and transparent approach to the inclusion and exclusion process [23].

### 2.3. Information Sources and Search

The literature search for relevant publications was conducted by mining four major electronic scholarly databases (PubMed/MEDLINE, Scopus, SPORTDiscus, and Web of Science) up to 20 January 2023. The best combination of keywords was achieved by means of literature familiarization through a preliminary search and reading of publications selected either using the PubMed “Medical Subject Headings” (MeSH) option or manually based on reference articles in this field of study. Various combinations of keywords and synonyms were employed in the title, abstract, or keywords field: (sarcopenia) AND (“aged” OR “elderly” OR “old”) AND (“high-intensity interval training” OR “HIIT” OR “high intensity interval” OR “interval training” OR “muscle strength”). Additionally, the reference lists of the identified studies were manually examined to identify potential studies that may not have been captured by the electronic searches. Furthermore, an external expert was consulted to verify the final list of references included in this review, ensuring comprehensive coverage. 

The screening process, which involved reviewing the title, abstract, and reference list of each study, was independently conducted by two authors (MPDM and JAML). The two authors also thoroughly reviewed the full versions of the included papers to ensure alignment with the selection criteria. A secondary search within the reference lists of the included records was performed to uncover any additional relevant studies. In cases of discrepancies in the selection process, a discussion took place between the two authors, and a third author (JPM) was consulted when needed. Potential errata for the included articles were duly considered. This rigorous approach aimed to comprehensively identify and include the relevant literature in the present review.

### 2.4. Data Extraction

The data extraction process adhered to the recommendations of the “Cochrane Consumers and Communication Review Group” and utilized their data extraction template [24], implemented using a Microsoft Excel sheet (version 2401, Microsoft Corporation, Redmond, WA, USA). The Excel sheet was specifically designed to evaluate inclusion criteria and systematically assess all selected studies. Both aspects of the process were independently executed by two authors (BMV andHIC). In cases where discrepancies arose regarding the eligibility of a study, discussions were held to reconcile differences. For transparency and documentation purposes, all full-text articles that were excluded from the review, along with the corresponding reasons for exclusion, were meticulously recorded in the Excel sheet.

### 2.5. Risk of Bias Assessment 

**Figure 2:**
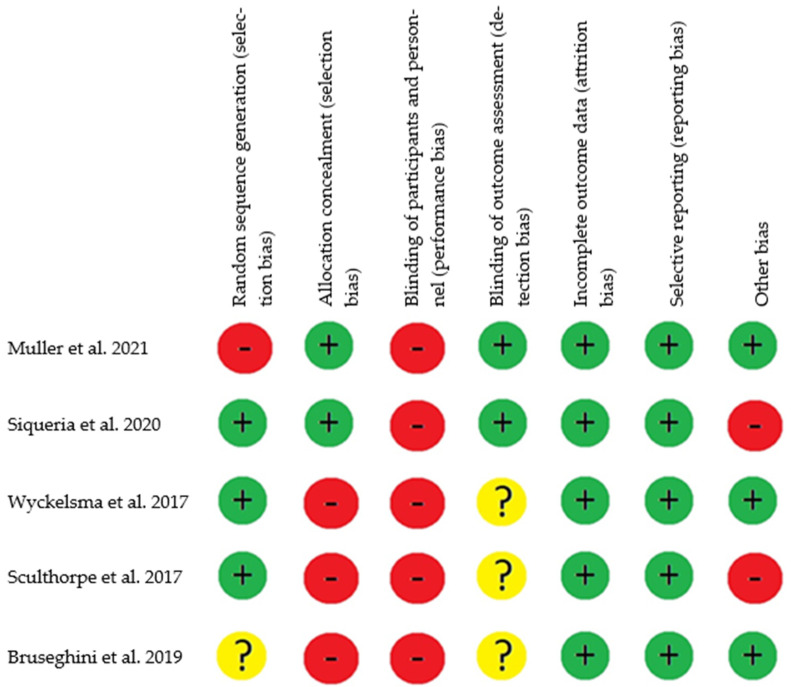
Risk of bias assessment based on the Cochrane tool [26,27,28,29,30]. Note: Low risk of bias +, high risk of bias –, and unclear risk of bias?

To assess the risk of bias in the included studies, we utilized the tool recommended by the Cochrane manual for systematic reviews, designed specifically for evaluating Randomized Controlled Trial (RCT) interventions. This tool provides a reliable assessment of various domains within the studies, with each domain being categorized as having a high, low, or unclear risk of bias [25]. Among the five manuscripts assessed—Müller et al. [26], Siqueira et al. [27], Wyckelsma et al. [28], Sculthorpe et al. [29], and Bruseghini et al. [30]—each study was found to have a “high risk of bias” in at least one domain [25] (Figure 2). In the domain of random sequence generation (selection bias), only one study was categorized as having a “low risk of bias” [26], while another study was marked as “unclear risk” [30]. The remaining articles provided sufficient information to ascertain the adequacy of the randomization sequence generation process, resulting in an overall assessment of “low risk” for this domain.

Concerning allocation concealment (selection bias), in only two studies, concealment of the sequence performed was adequate. The randomization process for the recruitment of participants was carried out by researchers who did not participate in the intervention, thus obtaining a “low risk” [26,27]. For the other studies, “high risk” was obtained, since the process of generation of the allocation sequence was not sufficiently described [28,29,30]. 

Regarding the blinding of participants and personnel, none of the studies implemented blinding for participants and personnel [26,27,28,29,30]. Concerning the blinding of outcome assessment (detection bias), two studies reported blinding of evaluators, resulting in a “low risk” [26,27]. The remaining studies lacked sufficient information on the blinding of evaluators, leading to an assessment of “unclear risk” [28,29,30]. Concerning incomplete outcome data (attrition bias), all of the studies reported their results and obtained a “low risk” [26,27,28,29,30]. In terms of selective reporting (reporting bias), all studies provided comprehensive reporting of estimates, variability, and intervention protocols, resulting in a “low risk” of reporting bias [26,27,28,29,30]. Finally, concerning other biases, two studies, when analyzing biases through intention-to-treat, were categorized as “high risk” [28,30]. The remaining studies were deemed free of other sources of bias, attaining a “low risk” [28,29,30]. 

Two authors (BMV and HIC) independently conducted the screening and assessment of the included articles. Discrepancies were resolved through consensus between the two authors, obviating the need for intervention by a third or fourth author (JPM, NLB).

### 2.6. Methodological Quality Assessment 

To assess the methodological quality of the studies included in this systematic review, the PEDro scale, utilizing a numerical scoring system ranging from 0 to 10, was employed. A summary of the assessment and scores for each study in different domains is provided below.

Regarding the assessment, we noted the following:

- All studies demonstrated an adequate generation of the sequence [26,27,28,29,30].
- Only one study had an adequate concealment of the allocation sequence [26].
- All studies exhibited baseline comparability [26,27,28,29,30].
- No study implemented blinding for participants and personnel [26,27,28,29,30].
- Only two studies blinded the evaluator [27,29].
- Adequate follow-up was reported in two studies [27,29].
- Intention-to-treat analysis was conducted in two studies [27,29].
- All studies included between-group comparisons [26,27,28,29,30].
- Similarly, all studies provided appointed estimates and variability [26,27,28,29,30].

The evaluation of the selected studies, as conducted through a scoring system, yielded the following scores for each respective study:

- Müller et al. [26]: score of 7/10;
- Siqueria et al. [27]: score of 6/10;
- Wyckelsma et al. [28]: score of 5/10;
- Sculthorpe et al. [29]: score of 6/10;
- Bruseghini et al. [30]: score of 6/10.

## 3. Results

### 3.1. Study Identification and Selection

**Figure 3:**
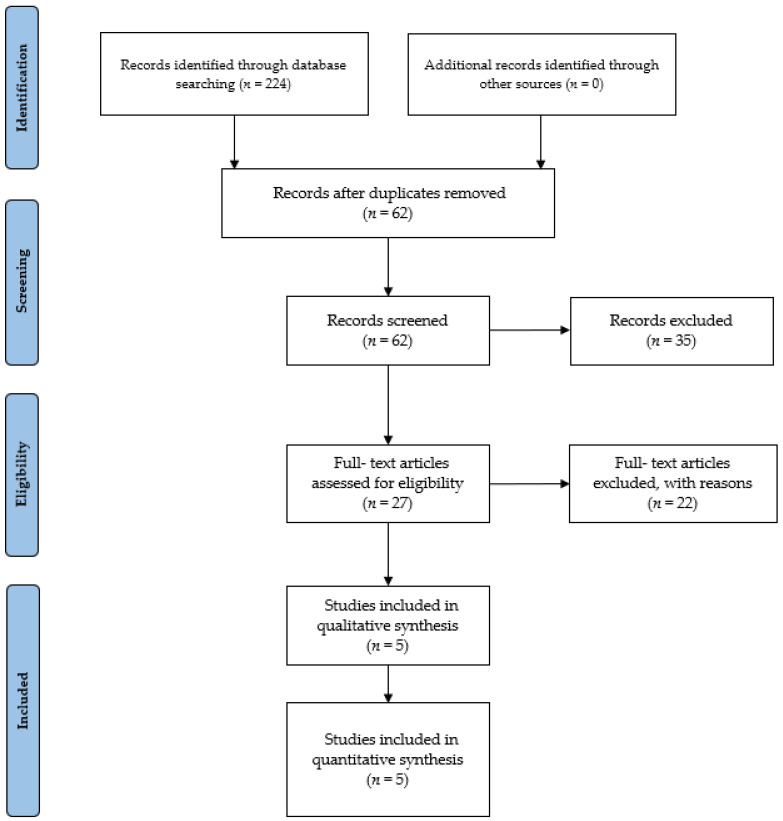
PRISMA 2020 flow diagram.

The initial database search yielded a total of 224 items. Subsequently, these studies were imported into reference management software (EndNoteTM X9, Clarivate Analytics, Philadelphia, PA, USA). Removal of duplicates, either through automated or manual processes, resulted in the elimination of 62 references. Thirty-five articles were removed for being irrelevant, based on titles and abstracts. The remaining 27 studies were selected for in-depth reading and analysis. After a comprehensive examination of the full texts, 22 studies that did not meet the eligibility criteria (*n* = 2 not meeting population criteria, *n* = 1 not meeting the comparator criteria, *n* = 7 not meeting the intervention criteria, *n* = 12 not meeting the study design criteria) were excluded. Finally, five studies meeting the criteria were incorporated into our review study (see Figure 3 for a visual representation of the selection process).

### 3.2. Study Characteristics and Context

The studies analyzed regarding HIIT in older adults reveal significant diversity in the demographic and physical characteristics of participants, with an average age ranging from 65.8 to 69.4 years and a range of 60 to 75 years. The sample size ranged from 12 to 41 participants, with studies showing a male predominance. In terms of physical characteristics, an average body weight of 77.8 kg, a mean height of 172 cm, and an average body mass index (BMI) of 26.5 are highlighted. The initial physical condition of participants varies, with some being active with a physical activity frequency of 2.6 h per week. The average duration of training sessions fluctuates between 30 and 50 min, with a frequency of 2 to 3 days per week and a total of 24 to 36 sessions during the intervention period. Specific exercises encompass a variety ranging from traditional strength exercises (leg press, knee flexion) to aerobic activities such as running. Exercise intensity is adjusted using percentages of 1RM, maximum heart rate (HRmax), or RPE. Overall, interventions are supervised by trainers, and pre- and post-intervention measurements are conducted to assess functional capacity, cardiorespiratory function, body composition, and neuromuscular activation, among others, using various measurement methods such as functional tests, cycle ergometers, DXA, and muscle biopsies. The reviewed results are statistically significant (*p* < 0.05) across various areas following training interventions in older adults. Significant improvements are observed in functional capacity, with a 15.9% increase in Sit-to-Stand at 16 weeks, along with enhancements in Timed Up and Go and stair climbing tests. Regarding cardiorespiratory capacity, there are significant increases in maximum power (11.2%) at 16 weeks, as well as in peak VO_2_ (16.5%) and cycling economy. Additionally, significant improvements are evident in body composition, including increases in lean mass and reductions in body fat. Neuromuscular capacity experiences statistically significant improvements, such as increases in muscle activation and isometric quadriceps strength, with a 7% increase at 90° knee flexion. In terms of performance, there are significant improvements in maximum effort work, time to exhaustion, and maximum power. Furthermore, notable physiological changes, such as increases in muscle pennation angle and quadriceps activation, are highlighted. In summary, the results underscore that various training modalities, by achieving statistically significant improvements, can provide comprehensive benefits in older adults in terms of functional capacity, cardiorespiratory function, body composition, neuromuscular performance, and physiological aspects.

Characteristics of the included studies as well as the details of HITT protocols can be found in Table 2.

## 4. Discussion

In an aging society, the burden imposed by sarcopenia is increasingly marked, both from an epidemiological and clinical perspective. Muscle mass decline is a physiological process that starts from the early age of 30 (where the decrease can be approximately computed at 3–8% per decade) and significantly increases after the age of 60 [31]. This process is even more accelerated in patients with sarcopenia. However, its detrimental impacts extend beyond muscle loss and related muscle tissue changes, affecting the entire health-related perceived quality of life and well-being. Indeed, several co-morbidities are associated with sarcopenia, ranging from dysmetabolic impairments to chronic degenerative disease.

As such, there is an onus to develop preventative strategies that can be effectively applied to old and frail populations to mitigate against the burden generated by sarcopenia [32]. Among non-pharmacological interventions, HIIT has gained increasing popularity in recent years for its potential benefits on muscle strength, endurance, power, and overall health, and it appears to be a valuable exercise strategy for older adults, even though most research has been conducted on healthy populations and in young and middle-aged adults. Little is known about senior populations, in which the impact of HIIT has been generally explored on metabolic and cardiovascular disease [33]

The purpose of this systematic review was to evaluate the effects of HIIT on muscle strength for the prevention and treatment of sarcopenia in older adults and to establish programming parameters for optimal exercise prescription. We also wanted to assess the safety of this type of exercise in this specific population in light of previous suggestions that it could be unsuitable [11,34,35]. Because of the lack of studies examining the effects of HIIT in older adults, the effects of this type of exercise on increasing muscle strength and function, as well as its general safety, are unclear. The main finding of our analysis is that HIIT is effective in increasing muscle strength and in the prevention and treatment of sarcopenia in older adults, with a moderate main effect being found. This complements a previous review that supported the use of high-impact exercise for the improvement of muscle strength [34,36]. Other meta-analyses [11,37] have described the benefits of HIIT for the prevention and treatment of sarcopenia in older adults.

HIIT has previously been shown to promote a number of metabolic adaptations [38,39]. While much is known about aerobic adaptations to HIIT, complete characterization of skeletal muscle remodeling, as a result of this type of training is unclear. The majority of HIIT protocols, including those reviewed in the present study, have utilized the cycling exercise modality which primarily loads the leg musculature. Varying HIIT protocols lasting between 3–15 weeks have resulted in modest increases in total body and trunk lean mass [40,41,42]. Muscle hypertrophy is dependent upon an increase in the ratio of myofibrillar protein synthesis and breakdown [40]. Although the studies overviewed in the present review did not directly measure muscle protein synthesis, a previous study by Scalzo et al. [43] demonstrated an increase in mitochondrial biogenesis and muscle protein synthesis in the vastus lateralis after nine cycling sessions of interval training at a resistance equivalent to 7.5% of body mass for 4–8 bouts of 30 s. Similarly, Di Donato et al. [44] demonstrated a significant increase in mitochondrial and myofibrillar protein synthesis 24–28 h postexercise following a higher intensity continuous exercise bout (60% Watt_max_) compared to a lower intensity bout (30% Watt_max_). Damas et al. [45] observed a significant correlation between myofibrillar protein synthesis and hypertrophy in the early stages of resistance training. However, at 3 weeks, following five resistance-training bouts, muscle hypertrophy was no longer correlated with muscle damage but with protein synthesis.

Researchers have also observed that even in older adults and in mobility-limited subjects, power training resulted in increased gait speed which was attributed to improvements in voluntary muscle activation [46]. Muscle mass, strength, and power decline through the aging process, and neuromuscular function can deteriorate with age through disuse, particularly of type II muscle fibers [47]. In their review of sarcopenia and dynapenia research, Mitchell et al. [48] determined that from a peak in the third decade of life for men and fourth decade of life for women, the mean rate of muscle mass loss is 0.47%∙yr^−1^ for men and 0.37%∙yr^−1^ for women, though in the eighth decade the rate of loss accelerated to 0.8–0.98%∙yr^−1^ and 0.64–0.7%∙yr^−1^, respectively. By age ~65 years, muscle power, an important component of functional movement, declines at a rate of ~3.5%/yr, nearly twice as quickly as strength [49,50]. Decreases in muscle mass and muscle size of predominantly type II muscle fibers have been associated with increased age [50]. The mechanisms, however, are not fully understood.

Strategies to prevent the age-related decline in cardiorespiratory fitness (CRF) and muscular performance may help to prevent or slow the progression of sarcopenia and its associated functional declines in generally healthy older adults. Even though the World Health Organization [51] recommends concurrent endurance (>150 min/wk) and resistance training (>2 sessions/wk), a lack of free time is a major barrier to attaining these exercise goals [52]. In this regard, several small, randomized trials utilizing less time-consuming HIIT protocols, characterized by brief intermittent bouts of high-intensity aerobic exercise, have emerged over recent years and revealed impressive effects on cardiovascular health and CRF. However, Ferreira et al. [53], in a cross-sectional study, showed that being involved in an aerobic training program versus being sedentary did not affect the sarcopenia prevalence among older women. In contrast, Lui et al. [11] in their narrative review, showed that HIIT might become a promising potential method for treating sarcopenia in older adults and obtaining remarkable metabolic changes in sarcopenia patients. Firstly, acute skeletal muscle responses can occur. HIIT upregulates 22 mitochondrial genes in older people, including genes participating in translational regulation and mitochondrial tRNA transferase, thereby resulting in a significant increase in protein abundance [54]. In this context, HIIT induces great growth of muscle, prevents skeletal muscle atrophy, and improves motor function via promoting great phosphorylation of the mammalian target of rapamycin (mTOR) and ribosomal protein S6 kinase (rps6) and inducing the expression of transcriptional coactivator peroxisome proliferator-activated receptor γ coactivator 1α (PGC-1α), which is crucial for mitochondrial biogenesis [55,56]. It is also of importance in the vascularization of muscle [57]. 

Collectively, HIIT significantly improved muscle strength [34,35] and reduced fat in the blood and liver [58]. Nevertheless, HIIT may not reduce body weight while reducing body fat because of muscle hypertrophy [35]. For instance, a recent study summarized the molecular mechanisms of HIIT in the treatment and prevention of sarcopenia [11]. However, despite the positive effects found in this review, some limitations should be acknowledged. First, the limited number of studies available and the high risk of bias severely limit the validity and generalization of the findings as well as the overall strength of evidence. Second, the heterogeneity in the characteristics of the older adults and populations across the included studies, such as age, baseline health status, and comorbidities, may impact the generalizability of the findings to other groups different from the specific subgroups of older adult individuals overviewed here. Third, other lifestyle factors, such as diet, medication use, and adherence to exercise regimens, were not consistently reported in all studies. These factors may act as confounders, making it challenging to isolate the specific impact of HIIT on muscle strength in sarcopenic older adults. Fourth, the absence of a universally accepted definition and diagnostic criteria for sarcopenia could introduce variability in participant selection across studies, potentially influencing the outcomes reported in this systematic review. 

The insights derived from this systematic review offer several practical applications for healthcare and exercise practitioners in the context of preventing and treating sarcopenia in older adults through HITT. 

Overall, the body of evidence supporting the positive effects of HIIT on muscle strength can inform the development of comprehensive geriatric care plans. Healthcare providers and geriatricians may consider integrating HIIT as a structured component of rehabilitation or maintenance programs for older adult individuals to mitigate the progression of sarcopenia and enhance overall functional capacity. Exercise practitioners play a pivotal role in implementing evidence-based strategies to enhance muscle strength and mitigate sarcopenia in older adults, and they can use the evidence from this systematic review to develop individualized exercise prescriptions for older adult clients at risk of sarcopenia. Tailoring HITT protocols based on factors such as baseline fitness, health status, and mobility can optimize the benefits of exercise while considering individual limitations and preferences. Moreover, this systematic review suggested that HIIT can be effective in improving muscle strength among older adults. Exercise practitioners can design progressive and structured HIIT programs that gradually increase intensity, duration, and frequency over time. This approach ensures that older adult clients can safely adapt to and benefit from the demands of HIIT while minimizing the risk of injury. Additionally, incorporating functional movements into HIIT sessions is crucial for enhancing not only muscle strength but also overall functional capacity in daily activities. Exercise practitioners should focus on exercises that mimic real-life movements, promoting the transfer of strength gains to functional tasks. 

Lastly, this systematic review identified gaps in the existing literature, such as the need for larger sample sizes, higher-quality, long-term studies involving also female subjects and consensus on diagnostic criteria for sarcopenia. This can inform future research directions, encouraging investigators to design well-controlled, longitudinal studies that explore the sustained impact of HIIT on muscle strength, and address methodological inconsistencies across trials.

In summary, while older adults can feasibly engage in HIIT, it should be approached with caution and individualization. Consulting with healthcare professionals and fitness experts, considering the individual’s health status, and gradually progressing the intensity can contribute to a safe and effective HIIT program for older adults. Moreover, older adults may feasibly and longitudinally engage in HITT with appropriate modifications and guidance. While the type and intensity of physical activity may vary based on individual health and fitness levels, research has shown that HITT can improve cardiovascular health, strength, and flexibility in older adults. However, it is essential to consult with a healthcare provider or certified fitness professional before starting any new exercise program, especially for older adults with underlying health conditions or injuries. Additionally, modifications such as slower movements, shorter intervals, and longer rest periods may be necessary to accommodate age-related changes in mobility and balance. Regularly monitoring progress and adjusting the program as needed can also help ensure safety and effectiveness over time.

## 5. Conclusions

The findings of this review confirm the feasibility and the effectiveness of HIIT-based protocols in old populations, showing their positive impacts on a variety of variables, including body composition, muscle quality, cardiorespiratory, neuromuscular, and performance capacity. Considering the above information, HIIT can be a viable strategy for improving muscle strength and countering sarcopenia in older adults. However, given the limited number of studies included in the present review and their shortcomings, further research is warranted. In particular, besides addressing these limitations, future studies should investigate the feasibility of implementing individualized approaches to provide proper guidance, ensure safety, and maximize the benefits of HIIT for this vulnerable population.

## Figures and Tables

**Table 1:** Inclusion and exclusion criteria according to the “Population, Intervention, Comparison/Comparator, Outcome, Study design” (PICOS) approach.

	Inclusion Criteria	Exclusion Criteria
**Population**	Populations without any injury or illness, with normal vision, and no history of neuropsychological impairment and/or other special conditions	Studies carried out with animals. Populations with special conditions (some types of pathology other than sarcopenia, type II diabetes, cardiovascular pathology, and obesity). Populations aged less than 65 years old.
**Intervention**	Studies with high-intensity interval training protocols studying the effects of strength exercises designed with an intervention group and a control group	Studies with high-intensity interval training protocols combined with another type of intervention that could mask the results of the former intervention (either another type of training, nutritional, or pharmacological ergogenic aids).
**Comparison/comparator**	Passive control conditions	Intervention conditions other than passive conditions.
**Outcome**	Muscle strength improvements	Physiological or physical conditions not related to the included outcomes.
**Study design**	Counterbalanced cross-over design (either randomized or non-randomized since none of them reveals significant differences in control conditions)	Study designs that do not allow within-subjects comparisons for the two conditions.
**Additional criteria**	Only indexed, original, full-text studies	Articles other than original research (e.g., reviews, letters to editors, trial registrations, proposals for protocols, editorials, book chapters, and conference abstracts).

**Table 2:** Characteristics of the included studies.

**Müller et al. [26]**																
**Population**	***Participants***	***Age (Years)***	***Sex***	***Body Mass (Kg)***	***Height (Cm)***			***BMI***			***Sample***	***Exclusion Pathologies***	***Assistance***			
	35	65.8 ± 3.9	M	89.3 ± 9.9	172.4 cm ± 7.4			18.75			TST + HIIT: N = 18	Neuromuscular, cognitive, metabolic, hormonal, cardiovascular, smokers.	100%			
				84.7 ± 14.8							TPT + HIIT: N = 17					
				85.3 ± 12.8							HIIT: N = 35					
**Intervention**	***Duration (Week)/*** ***Frequency (Day)***	***Ig***	***Cg***	***Intensity/Velocity***	***Phase/Time***			***Characteristics***			***Measurement***	***Exercise***	***Questionnaire***			
	16/2	TST + HIIT	No	65–80% RM	2 s (concentric and eccentric)			Week 1–4: 2 x (12–15 rep 65% RM x 180 s rest) Week 13–16: 4 x (6–8 rep 80% RM x 180 s rest)			Functional capacity	Sit-to-Stand (>n° rep x 30 s), Timed- Up and Go, climbing stairs (16 cm)	-			
		TPT + HIIT		40–60% RM	2 s eccentric and maximum concentric			Week 1–4: 3 x (8 rep 40% RM x 180 s rest) Week 13–16: 4 x (6 rep 60% RM x 180 s rest)			Cardiorespiratory capacity	Peak power in cycling: Wmax, VO_2_max, cycling economy				
		HIIT		75–90% VO_2_max	-			Cycle-ergometer: Warming 5 min 60–65% HRmax Cadence 70–75 rpm Week 1–4: 3 x (4 min 75–85% HRmax x 2 min active rest) Week 13–16 3 x (4 min 85–90% HRmax x 2 min active rest)			Body composition (DEXA)	Before intervention, 8 weeks, 16 weeks: body fat mass, lean body mass, total body fat mass, total lean body mass				
**Results**	***Measurement***		***Exercise/Parameter***		***TST***							***TPT***				
	Functional capacity		Sit-to-Stand		8 weeks: ↑ 13.3% (*p* < 0.001) ES = 0.91 16 weeks: ↑ 15.9% (*p* < 0.001)							8 weeks: ↑ 30% (*p* < 0.001) ES = 1.46 16 weeks: ↑ 27.1% (*p* < 0.001)				
			Timed Up and Go		8 weeks: (*p* < 0.001) No differences ES = 0.47. 16 weeks: ↑ 10% (*p* < 0.001)							16 weeks: ↑ 14% (*p* < 0.001) ES = 0.54				
			Climbing stairs		8 weeks: ↑ 13.2% (*p* < 0.005) 16 weeks: ↑ 15.2% (*p* < 0.001) ES = 0.54							8 weeks: ↑ 13.9% (*p* < 0.005) 16 weeks: ↑ 12.3% (*p* < 0.001) ES = 0.78				
	Cardiorespiratory capacity		Wmax		8 weeks: ↑ 6.7% (*p* < 0.001) 16 weeks: ↑ 9.9% (*p* < 0.001) ES = 0.75							8 weeks: ↑ 8% (*p* < 0.001) 16 weeks: ↑ 11.1% (*p* < 0.001) ES = 1.17				
			VO_2_max		8 weeks: ↑ 7.8% (*p* < 0.005) 16 weeks: ↑ 10% (*p* < 0.001) ES = 0.53							8 weeks: ↑ 22.3% (*p* < 0.005) 16 weeks: ↑ 37.8% (*p* < 0.001) ES = 0.54				
			Cycling economy		8 weeks: ↑ 9.9% (*p* < 0.001) 16 weeks: ↑ 9.5% (*p* < 0.001) ES = −0.6							8 weeks: ↑ 10.7% (*p* < 0.001) 16 weeks: ↑ 17.2% (*p* < 0.001) ES = 0.34				
**Siqueria et al. [27]**																
**Population**	***Participants***	***Age (Years)***	***Sex***	***Body Mass (Kg)***	***Height (cm)***		***BMI***				***Sample***	***Exclusion Pathologies***	***Assistance***			
	41	63.9 ± 2.5	F	74.4 ± 14.7	153.4 cm ± 4.3		31.5				CG: n = 21	Cardiovascular disease and osteo-articular restrictions for exercising.	CG: 82.6%			
		64.8 ± 3.6		74.4 ± 14.7	155.1 cm ± 5.8		30.1				IG: n = 20		IG: 82.2%			
**Intervention**	***Duration (Week)/*** ***Frequency (Day)***	***IG***	***CG***	***Intensity/Velocity***	***Phase/Time***		***Characteristics***				***Measurement***	***Exercise***	***Questionnaire***			
	12/2	CG	No	Borg Scale (6–20) RPE: 13–16/-	2 s (concentric and eccentric)		Week 1–4: 36 min RPE: 13 Week 5–8: 36 min RPE: 14 Week 9–10: 36 min RPE 15 Week 11–12: 36 min RPE 16				Cardiorespiratory capacity	Stationary running, front kick and cross-country skiing Aquatic intervention	Informed consent form			
		IG		RPE: 11–18/-	2 s (concentric and eccentric)		Week 1–4: 9 rep x (2 min RPE:16 + 2 min RPE 11) Week 5–8: 12 x (1.5 min RPE:17 + 1.5 min RPE 11) Week 9–10: 18 x (1 min RPE:18 + 1 min RPE 11) Week 11–12: 18 x (1 min RPE:18 + 1 min RPE 11)				Neuromuscular capacity					
**Results**	***Measurement***	***Exercise Parameter***			***CG***							***IG***				
	Cardiorespiratory capacity	Cycle-ergometer RHR (bpm)			↓ 15.4% (*p* < 0.05) Pre CG: 83 ± 21 Post CG: 75 ± 11							↓ 11.8% (*p* < 0.05) Pre: 80 ± 14 Post: 75 ± 14				
		VO_2_max (mL·kg^−1^·min^−1^)			↑ 10.3% (*p* < 0.05) Pre: 26.30 ± 3.68 Post: 28.76 ± 5.05							↑ 16.5% (*p* < 0.05) Pre: 24.07 ± 4.10 Post: 26.01 ± 7.95				
		Exhaustion tolerance			↑ 5.6% (*p* < 0.05) Pre: 13.08 ± 1.91 Post: 14.02 ± 1.88							↑ 13.5% (*p* < 0.05) Pre: 12.75 ± 1.45 Post: 14.04 ± 1.40				
	Neuromuscular capacity	Maximal dynamic strength (kg)			↑ 5.9% (*p* < 0.05) Pre: 30.92 ± 6.29 Post: 32.42 ± 6.42							↑ 12.5% (*p* < 0.05) Pre: 28.00 ± 5.68 Post: 29.50 ± 5.21				
		Dynamic resistance			↑ 15.3% (*p* < 0.05) Pre: 11.83 ± 2.21 Post: 13.08 ± 3.48							↑ 6.7% (*p* < 0.05) Pre: 12.63 ± 2.07 Post: 14.00 ± 2.62				
		Neuromuscular activation (uV)			↑ 38.4% (*p* < 0.05) Pre: 94.16 ± 40.82 Post: 102.30 ± 45.26							↑ 47.7% (*p* < 0.05) Pre: 121.00 ± 67.62 Post: 151.13 ± 72.62				
		Muscular thickness (cm)			↑ 4.8% (*p* < 0.05) Pre: 6.07 ± 0.79 Post: 6.35 ± 0.87							↑ 6.7% (*p* < 0.05) Pre: 6.26 ± 0.98 Post: 6.62 ± 1.11				
		*Muscular volume (cm^3^)*			↑ 68.2 cm^3^ Post-IRT compared to Pre-IRT (*p* = 0.001)							↑ 42.2 cm^3^ Post-HIIT compared to Pre-HIIT (*p* = 0.003)				
		*ACSA in relation to IMAT intermuscular adipose tissue:*			50% LF ↓ Post-IRT compared to Pre-IRT (*p* = 0.008) 75% LF ↓ Post-IRT compared to Pre-IRT (*p* = 0.001)							50% LF ↓ Post-HIIT compared to Pre-HIIT (*p* = 0.001) CI: 95%; 75% LF: No significance (*p* > 0.05)				
		Muscular torque			IRT ↑ 7.8% TMVC: 90° ↑ 11.5 N·m ± 17.1 (*p* = 0.040) IRT ↑ TC: 120° s^−1^ ↑ 8.8 N·m ± 13.0 (*p* = 0.008)							HIIT no significance				
		*Pennation angle, PCSA and specific torque*			PCSA 50% LG: ↑ Post-IRT compared to Pre-IRT (*p* = 0.025) Significant effect time-training IRT ↑ Post-IRT compared to Pre-IRT (*p* = 0.004)							Significant effect time-training IRT ↑ Post-HIIT compared to Pre-HIIT (*p* = 0.001)				
		Specific isometric strength (Strength/ACSA)			With IMAT torque·cm^2^ from ACSA it remained unchanged Without IMAT torque·cm^2^ from ACSA: Pre-IRT (63.8 N·cm^−2^ ± 5.6) Post-IRT (63.0, N·cm^−2^ ± 9.1)							With IMAT torque·cm^2^ from ACSA it remained unchanged Without IMAT torque·cm^2^ from ACSA: Pre-HIIT 66.4 N·cm^−2^ ± 6.1; Post-HIIT 60.8 N·cm^−2^ ± 7.5				
		*Neuromuscular activation*			↑ Post-IRT compared to Pre-IRT (*p* = 0.011)							-				
**Wyckelsma et al. [28]**																
**Population**	***Participants***	***Age (Years)***	***Sex***	***Body Mass (Kg)***	***Height (cm)***	***BMI***				***Sample***		***Exclusion Pathologies***	***Assistance***			
	Beginning: 15	69.4 ± 3.5	6 M 2 F	75.2 ± 13.0	170.8 ± 10.4 cm	21.6				IG: *n* = 8		Type I or type II diabetes, chronic heart disease, severe hypertension, severe overweight/obesity, uncontrolled metabolic disease, cardiovascular disease and injuries.	IG: 83% (30/36 sessions)			
	Intervention: 13		3 M 4 F							CG: *n* = 7						
**Intervention**	***Duration (Week)/Frequency (Day)***	***IG***	***CG***	***Intensity/Velocity***	***Phase/Time***	***Characteristics***				***Measurement***		***Exercise***	***Questionnaire***			
	12/3	IG	No	RPE 17/-	**-**	Warming: 5 min cycle-ergometer PP: 4 rep x (4 min 90–94%HRmax x 4 min active resting 50–60% HRmax), Calm down: 5 min cycle-ergometer				Cardiorespiratory capacity Plasma changes (K+), union of (3H) muscular ouabain and NKA isoforms		Cycle-ergometer	-			
**Results**	***Measurement***	***Exercise Parameter***			***CG***							***IG***				
	Cardiorespiratory capacity	HR peak (b x min^−1^)			Pre: 141 ± 11 Post: 142 ± 14							↑ 6.8% (*p* < 0.05) Pre: 136.2 ± 16.4 Post: 144.3 ± 14.4				
		VO_2_peak (mL x kg^−1^ x min^−1^)			Pre: 23.6 ± 5.3 Post: 23.8 ± 5.3							↑ 16.2% (*p* < 0.05) Pre: 24.7 ± 5.4 Post: 28.7 ± 5.1				
	Performance capacity	WRpeak (W)			Pre: 142.0 ± 46.4 Post: 147.1 ± 40.2							↑ 25.23% (*p* < 0.05) Pre: 145.0 ± 49.5 Post: 181.2 ± 52.4				
		Work (J)			Pre: 47.914 ± 24.408 Post: 47.486 ± 24.834							↑ 60.46% (*p* < 0.05) Pre: 43.725 ± 21.282 Post: 70.050 ± 31.834				
		Time to RPE-17 (min)			Pre: 9.6 ± 3.1 Post: 9.7 ± 3.1							Pre: 7.3 ± 3.7 Post: 9.4 ± 4.5				
	Physiological capacity	[K+ ]v peak (mmol.L^−1^)			Pre: 4.88 ± 0.33 Post: 4.90 ± 0.42							↑ 10% (*p* = 0.056) Pre: 4.74 ± 0.41 Post: 5.23 ± 0.57				
		Δ[K+ ]v x work^−1^ (nmolx L^−1^ x J^−1^)			Pre: 22.3 ± 10.9 Post: 21.1 ± 14.7							(*p* > 0.05) No differences Pre: 21.4 ± 10.6 Post: 17.4 ± 4.5				
**Sculthorpe et al.** [29]																
**Population**	***Participants***	***Age (Years)***	***Sex***	***Body Mass (Kg)***	***Height*** ***(cm)***	***BMI***			***Sample Number***			***Exclusion Pathologies***		***Assistance***		
	33	62.3 ± 4.1	M	89.9 ± 17.1	175 ± 5.2	29.4			HITT: *n* = 22			-		100%		
		61.6 ± 5.0		87.5 ± 14.3	173 ± 5.5	29.1			CG: *n* = 11							
**Intervention**	***Duration (Week)/*** ***Frequency (Day)***	***IG***	***CG***	***Intensity/Velocity***	***Phase/Time***	***Characteristics***			***Measurement***			***Exercise***		***Questionnaire***		
	6/5	Conditioning	No	Week 1–2: 55% HRR/- Week 3–4: 60% HRR/- Week 5–6: 65% HRR/-	**-**	Perform the activity of participant’s preference, taking into account duration, HR and recommended intensity.			Body composition, static equilibrium and start point			Walking, jogging, cycling		PAR-Q		
	6/1.4	HIIT	No	40–50% PP 90% HRR	**-**	Warming: 5 min cycle-ergometer PP: 6 rep sprints x (30 s 50% PP90% HRR x 3 min active resting)						Cycle-ergometer		IPAQ		
**Results**	***Measurement***	***Exercise Parameter***			***CG***							***IG***				
	Performance capacity	WRpeak (W)			Phase A: 655 W Phase B: 661 W Phase C: 657 W Phase A–B: ↑ 1% ↑ 6 W (*p* > 0.05) Phase B–C: ↓ 0.6% ↓ 4 W (*p* > 0.05) Phase A–C: ↑ 2 W (*p* > 0.05)							Phase A: 699 W Phase B: 706 W Phase C: 831 W Phase A–B: ↑ 1% ↑ 7 W (*p* > 0.05) Phase B–C: ↑ 17.6% ↑ 125 W (*p* < 0.01) Phase A–C: ↑ 132 W (*p* < 0.01) Phase A: IG ↑ 44 W (↑ 6.7%) compared to CG Phase B: IG ↑ 44.7 W (↑ 6.7%) compared to CG Phase C: IG ↑ 173.8 W (↑ 26%) compared to CG				
		Relative WRpeak (W/Kg)			Phase A–B: No improvement (*p* > 0.05) Phase B–C: No improvement (*p* > 0.05)							Phase A–B: No improvement (*p* > 0.05) Phase B–C: ↑ 1.53 W/Kg ↑ 14% (*p* < 0.01) Phase C: IG ↑ 1.67 W/Kg (↑ 15%) compared to CG				
	Body composition	Total body mass			No effect PP on phase or, group (*p* > 0.05)							No effect PP on phase or, group (*p* > 0.05)				
		Total body lean mass			Phase A: 63.4 Kg Phase B: 63.7 Kg Phase C: 63.6 Kg Phase A–B: ↑ 0.4% (*p* > 0.05) Phase B–C: ↓ 0.1 (*p*> 0.05)							Phase A: 65.9 Kg Phase B: 66.1 Kg Phase C: 68.1 Kg Phase A–B: ↑ 0.3% (*p* > 0.05) Phase B–C: ↑ 3% (*p* < 0.05) Phase A: IG ↑ 2.5 Kg (↑ 3.9%) compared to CG Phase B: IG ↑ 2.4 Kg (↑ 3.7%) compared to CG Phase C: IG ↑ 4.5 Kg (↑ 7%) compared to CG				
		Total body fat mass			Phase A: 19.8 Kg Phase B: 19.9 Kg Phase C: 19.3 Kg Phase A–B: ↑ 0.1% (*p* > 0.05) Phase B–C: ↓ 0.7% (*p* > 0.05)							Phase A: 23.9 Kg Phase B: 22.8 Kg Phase C: 20.8 Kg Phase A–B: (*p* < 0.05) CI: 95% Phase B–C: (*p* < 0.05) CI: 95% Phase A: IG ↓ 4.1 Kg compared to CG Phase B: IG ↓ 2.9 Kg compared to CG Phase C: IG ↓ 1.5 Kg compared to CG				
**Bruseghini et al.** [30]																
**Population**	***Participants***	***Age (Years)***	***Sex***	***Body Mass (Kg)***	***Height*** ***(cm)***	***BMI***			***Sample Number***			***Exclusion Pathologies***			***Assistance***	
	12	69.3 ± 4.2	M	77.8 ± 10.4	172 ± 5.0	26.5			HIIT aerobic training: *n* = 12			Abnormal EKG, hypertension, cardiovascular, respiratory, metabolic, kidney failure, neurological, orthopaedics, anticoagulant treatment and antiplatelet therapy contraindication, drugs and alcohol abuse			100%	
									IRT: *n* = 12							
**Intervention**	***Duration (Week)/*** ***Frequency (Day)***	***IG***	***CG***	***Intensity/Velocity***	***Phase/Time***	***Characteristics***			***Measurement***			***Exercise***			***Questionnaire***	
	8/3	HIIT	No	85–95% VO_2_max/-	-	Warming:10 min cycle-ergometer PP: 7 rep x (2 min 85–95% VO_2_max x 2 min 40% VO_2_max)			Strength, mass, architecture and muscular quality, IMAT, and neuromuscular activation			Cycle-ergometer			IPAQ	
		IRT		Maximal concentric contraction		Warming: 10 min 3 rep x (7 submaximal knee extension) PP: 4 rep x (7 maximal concentric knee extension and eccentric knee flexion)						Cycle-ergometer Iso-inertial machine Yoyo Technology AB				
**Results**	***Measurement***	***Exercise Parameter***			***IRT***							***HIIT***				
	Neuromuscular capacity	ACSA (cm^2^)			25% LF ↑ IRT compared to HIIT (*p* = 0.024) 75% LF ↑ IRT compared to Post-HIIT (*p* = 0.08) 75% LF ↑ IRT compared to Pre-HIIT (*p* = 0.011) ↑ 4.47 cm^2^ Post-IRT compared to Pre-IRT (*p* = 0.001)							25% LF ↑ 3.19 cm (*p* = 0.001) 50% LF ↑ 3.03 cm (*p* = 0.005) 75% LF ↑ 3.40 cm (*p* = 0.004) ↑ 3.9 cm^2^ Post-HIIT compared to Pre-HIIT (*p* = 0.001)				
		Muscular volume (cm^3^)			↑ 68.2 cm^3^ Post-IRT compared to Pre-IRT (*p* = 0.001)							↑ 42.2 cm^3^ Post-HIIT compared to Pre-HIIT (*p* = 0.003)				
		ACSA in relation to IMAT intermuscular adipose tissue			50% LF ↓ Post-IRT compared to Pre-IRT (*p* = 0.008) 75% LF ↓ Post-IRT compared to Pre-IRT (*p* = 0.001)							50% LF ↓ Post-HIIT compared to Pre-HIIT (*p* = 0.001) CI: 95%; 75% LF: No significance (*p* > 0.05)				
		Muscular torque			IRT ↑ 7.8% MVC: 90° ↑ 11.5 N·m ± 17.1 (*p* = 0.040) IRT ↑ TC: 120° s^−1^ ↑ 8.8 N·m ± 13.0 (*p* = 0.008)							HIIT no significance				
		Pennation angle, PCSA and specific torque			PCSA 50% LG: ↑ Post-IRT compared to Pre-IRT (*p* = 0.025) Significant effect time-training IRT ↑ Post-IRT compared to Pre-IRT (*p* = 0.004)							Significant effect time-training IRT ↑ Post-HIIT compared to Pre-HIIT (*p* = 0.001)				
		Specific isometric strength (Strength/ACSA)			With IMAT torque·cm^2^ from ACSA it remained unchanged Without IMAT torque·cm^2^ from ACSA: Pre-IRT (63.8 N·cm^−2^ ± 5.6) Post-IRT (63.0, N·cm^−2^ ± 9.1)							With IMAT torque·cm^2^ from ACSA it remained unchanged Without IMAT torque·cm^2^ from ACSA: Pre-HIIT 66.4 N·cm^−2^ ± 6.1; Post-HIIT 60.8 N·cm^−2^ ± 7.5				
		Neuromuscular activation			↑ Post-IRT compared to Pre-IRT (*p* = 0.011)							-				

Notes: BMI: body mass index; IG: intervention group; CG: control group; HIIT: high-intensity interval training; TST: traditional strength training; TPT: traditional power training; Wmax: maximal workload; RPE: rate of perceived exertion; HRmax: maximal heart rate; RHR: resting heart rate; WRpeak: peak work rate; PP: peak power; LfHIIT: lower-frequency HIIT; PCSA: physiological cross-sectional area; IMAT: intermuscular adipose tissue; ACSA: anatomical cross-sectional area; PCSA: physiological cross-sectional area; IRT: iso-inertial resistance training; MVC: maximal voluntary contraction.

## Data Availability

No new data were generated.
